# Is Percutaneous Endoscopic Gastrostomy an Innocent Procedure? A Retrospective Single-Center Study

**DOI:** 10.3390/medicina61050802

**Published:** 2025-04-25

**Authors:** Ramazan Serdar Arslan, Yavuz Savas Koca, Semra Tutcu Sahin, Resad Beyoglu

**Affiliations:** 1 Department of General Surgery, Acipayam State Hospital, Acipayam 20800, Turkey; r.serdar.arslan@gmail.com; 2 Department of General Surgery, Servergazi State Hospital, Merkezefendi 20040, Turkey; yavuzsavaskoca@gmail.com; 3 Department of General Surgery, Faculty of Medicine, Celal Bayar University, Manisa 45000, Turkey; 4 Department of Emergency Medicine, Faculty of Medicine, Bandirma Onyedi Eylul University, Bandirma 10200, Turkey; rbeyoglu@gmail.com

**Keywords:** PEG, complications, risk factors, enteral nutrition, mortality, percutaneous endoscopic gastrostomy, early complication, late complication, buried bumper syndrome

## Abstract

*Background and Objectives*: Percutaneous endoscopic gastrostomy (PEG) is a safe, minimally invasive method preferred for long-term enteral nutrition. While most procedural complications are minor and occur in the early period, there are also major complications that can lead to death. This study aims to investigate the minor and major complications, the timing of minor complications in patients with PEG tube placement, and the relevant literature. *Materials and Methods*: We conducted a retrospective review of 652 patients who underwent PEG tube placement between 1 January 2010 and 31 October 2024. This study investigated the age, gender, primary disease, minor and major complications, early and late complications, and the time to the emergence of minor complications in patients who underwent PEG tube placement. *Results*: The majority of patients underwent PEG insertion due to neurological diseases, with stroke being the most common cause. The minor complication rate was 17.1%, while the major complication rate was 9.5%. The most common minor complication was peristomal infection (5.2%), and the most common major complication was buried bumper syndrome. Thirty-nine patients (12.7%) experienced complications in the early period, while 17 patients (5.5%) experienced them in the late period. The transverse colon, which was injured, was the most commonly affected internal organ. *Conclusions*: PEG is widely used for neurological diseases, such as cerebrovascular disease and dementia, with minor complications being more common than major ones. There is no significant difference between early and late minor complications.

## 1. Introduction

In patients unable to be fed orally, enteral or parenteral nutrition is employed to meet their metabolic requirements [1]. Parenteral nutrition incurs higher costs due to the risks associated with the intravenous route and the need for professional healthcare [1,2,3]. Enteral nutrition is vital for the continuity of mucosal functions in individuals with healthy gastrointestinal systems and for preserving intestinal flora and immunity. Moreover, enteral nutrition reduces the likelihood of bacteremia associated with bacterial translocation [1,2,3,4]. Enteral feeding can be provided via nasogastric or nasojejunal tubes, percutaneous endoscopic jejunostomy, surgical gastrostomy, surgical jejunostomy, radiological gastrostomy, or PEG [1,2,3,4].

PEG is the preferred method for long-term enteral nutrition in patients who cannot be fed orally and have normal gastrointestinal function. Indications for PEG include dysphagia due to neurological diseases, long-term coma, burns, cerebrovascular diseases, motor neuron diseases, cancer, inability to feed due to head and neck trauma, and mechanical obstruction of the laryngopharyngeal and oesophageal regions [4,5,6]. It can also be applied as a diversion to ensure safety in duodenal injuries and oesophageal anastomoses. The most commonly used PEG placement procedures are the ‘pull’ method defined by Gauderer [7] in 1980 and the ‘push’ method defined by Russell [8] in 1984. It is currently the gold standard for continuing enteral feeding because it can be performed at the patient’s bedside using sedation and/or local anaesthetics without requiring general anaesthesia, and it is comfortable with a low complication rate [9,10].

Although PEG-related complications are infrequent, the literature indicates that the rate of minor complications (tube dislocation, tube occlusion, leakage at the entry site, local wound infection, etc.) varies between 8 and 30%, while the rate of major complications (bleeding, peritonitis, perforation, necrotizing fasciitis, colocutaneous fistula, etc.) ranges from 1 to 4% [9,10,11,12,13]. This retrospective study aims to present the minor and major complications, the timing of minor complications in patients with PEG tube placement, and the relevant literature.

## 2. Materials and Methods

This study retrospectively reviewed 652 patients who underwent PEG tube placement in the Endoscopy Unit of the General Surgery Department at Servergazi State Hospital, either in the patient ward, intensive care unit, or palliative care unit, between 1 January 2010 and 31 October 2024. The patient’s age, gender, primary disease (indication for PEG), as well as minor and major complications and early and late complications that developed after the procedure, were investigated.

Complications that developed within the first two weeks after PEG tube placement were classified as early complications, while those that developed between two weeks and three months were classified as late complications. Data related to hospitalised patients were accessed through the hospital data system and archive files. For discharged patients, information was obtained by contacting the patients’ relatives or caregivers by phone.

Patients who had PEG catheters inserted at different institutions and then came to our hospital for outpatient or inpatient healthcare services were excluded from this study. Additionally, patients who had PEG placed by gastroenterologists in our hospital were also excluded. Commercial PEG kits (EzFeed, 18-20Fr, ZKSK^®^-Beijing, China) with standard features available in the hospital’s medical warehouse on the relevant date were used for the procedures.

### 2.1. Application Technique

The day before the PEG procedure, the relatives of the patients were provided with detailed information about this study, and informed consent forms were signed. The PEG procedure was also performed in the endoscopy unit for patients who could be transferred and whose general condition was stable and in the intensive care unit at the bedside for patients who were intubated, had a tracheostomy, or were difficult to move. The procedure was performed in the operating room for patients deemed at risk by the anaesthesiologist. The procedure was conducted after a minimum of eight hours of fasting.

First-generation cephalosporin was administered prophylactically 1–2 h before the procedure to patients who had not used antibiotics due to their primary disease. Peripheral oxygen saturation, electrocardiography (ECG), and systolic and diastolic blood pressures were continuously monitored during the procedure in all cases. Intravenous sedatives (midazolam 0.1 mg/kg and/or propofol 0.5–1 mg/kg) were administered to all cases by the anaesthesiologist. Oesophagogastroduodenoscopy was performed using fibre endoscopy (Fujinon^®^ Fujifilm EG 530, Tokyo, Japan). The presence of any pathology in the upper gastrointestinal system up to the second part of the duodenum that could prevent PEG was evaluated during endoscopy. After sufficient transillumination was achieved with gastroscopy or the puncture site was determined with finger waving, local anaesthesia was applied using prilocaine hydrochloride, and the guide wire was advanced to the stomach. The gastrostomy tube was extracted from the mouth with the assistance of a snare and subsequently positioned in the stomach. An 18–20 French standard PEG (EzFeed, ZKSK^®^-Beijing, China) set was used for the procedure. After positioning the PEG tube to ensure it could be pulled out freely, rotated around itself, and placed on the abdominal wall, the corresponding cm level from the skin was documented in the endoscopy note. The procedure was concluded after checking for bleeding. No leakage test was performed, and sterilisation conditions were maintained throughout the procedure.

### 2.2. Statistical Analysis

Statistical analysis was conducted using IBM SPSS Statistics for Windows, version 26.0 (IBM Corp., Armonk, NY, USA). Gender characteristics of the patients were presented as numbers and percentages, while their ages were presented as mean, standard deviation, minimum, and maximum values. The relationship between minor and major complications following PEG and diseases, as well as the relationship between early and late minor complications and diseases, were examined. Fisher’s exact test and the chi-square test were employed to determine differences between categorical variables. The statistical significance level in the study was accepted as *p* < 0.05 and *p* < 0.01, and analyses were performed accordingly.

## 3. Results

A total of 652 patient records with PEG applied were identified in the hospital’s automation system and archive files. Patients who could not be reached by phone (*n* = 46), those who could be reached but could not provide clear information (*n* = 54), and patients with only partially accessible data (*n* = 37) were excluded from this study. The investigation involved a thorough analysis of data from 515 patients. The examination of the gender distribution among the patients revealed that there were 218 males (42.4%) and 297 females (57.6%). The mean age of participants was 68.7 ± 15.2 years, with the youngest participant being 12 years old and the oldest 103 years old (Table 1).

**Figure 1:**
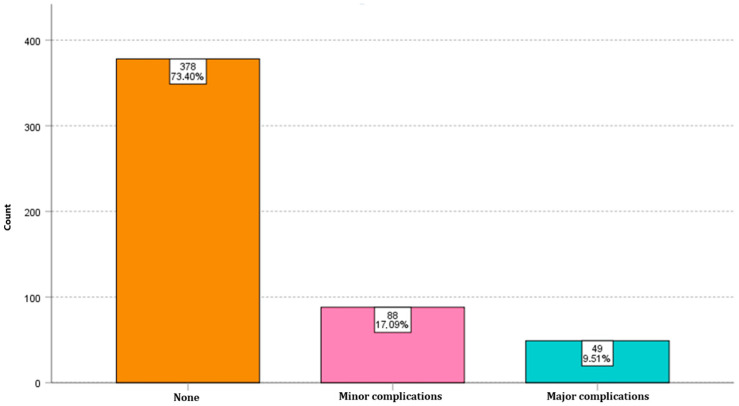
Number of minor and major complications.

No complications developed in 378 patients (73.4%). Minor complications were detected in 88 patients (17.1%), while major complications occurred in 49 patients (9.5%). Both minor and major complications were detected in 23 patients (4.4%). Thus, it was determined that 26.6% of the total patients developed complications (Figure 1). The analysis indicated that patients with neurological diseases comprised the majority of those who underwent PEG insertion (59.6%). PEG insertion was most commonly performed for cerebrovascular disease (stroke) (26%), followed by dementia (22.5%). Patients in intensive care constituted 15.1%, ranking third. Nasopharyngeal tumours were the most prevalent indication among malignancies, accounting for 3.4%.

The analysis of minor complications revealed that peristomal infection was the most prevalent, occurring in 5.2% of cases, while bleeding from the catheter edge was noted in 3.8% of cases. Seventeen patients (3.3%) experienced leakage from the PEG edge, while thirteen patients (2.5%) had the tube dislodged. Eleven patients, representing 2.1% of the total, exhibited pneumoperitoneum. The most common major complication after PEG was buried bumper syndrome, which occurred in 20 patients (3.8%). Aspiration pneumonia developed in 12 patients (2.3%), and bleeding was observed in 9 patients (1.7%). All patients with bleeding were treated gastroscopically, and haemodynamic stabilisation was achieved with supportive care. There were no cases requiring surgery due to major bleeding. Five patients (0.9%) had internal organ injuries, with four of these patients having a history of previous abdominal surgery. The most commonly injured organ was the transverse colon (four patients), and one patient sustained a small bowel injury.

**Figure 2:**
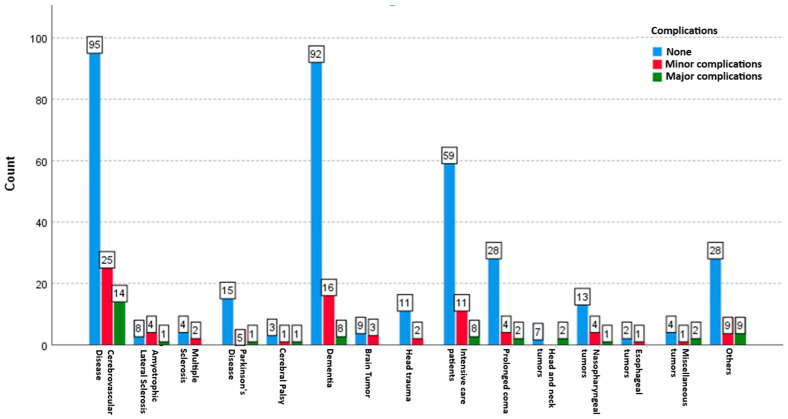
Analysis of the relationship between minor and major complications after PEG and diseases.

Necrotizing fasciitis was observed in three patients (0.5%). In patients who developed necrotizing fasciitis, the transverse colon was injured, leading to intra-abdominal multiple abscesses secondary to sepsis. A total of six patients died due to the PEG procedure (three patients with necrotizing fasciitis, two patients with aspiration pneumonia, and one patient who experienced cardiac arrest during the procedure), resulting in an observed mortality of 1.1%. Table 2 and Figure 2 present an analysis of the relationship between minor and major complications following PEG and associated diseases.

In neurological diseases, the difference in complications after PEG among patients with cerebrovascular disease, dementia, and Parkinson’s disease was significant (*p* < 0.05), while no statistical significance was found in patients with amyotrophic lateral sclerosis (ALS), multiple sclerosis (MS), cerebral palsy (CP), and brain tumours (*p* > 0.05). It was observed that the difference in post-PEG complications was observed in patients with head trauma, intensive care unit patients, and patients in long-term coma with reduced consciousness (*p* < 0.05). A statistical difference was found between oncological patients with nasopharyngeal tumours and other unclassified disease groups (*p* < 0.05).

**Figure 3:**
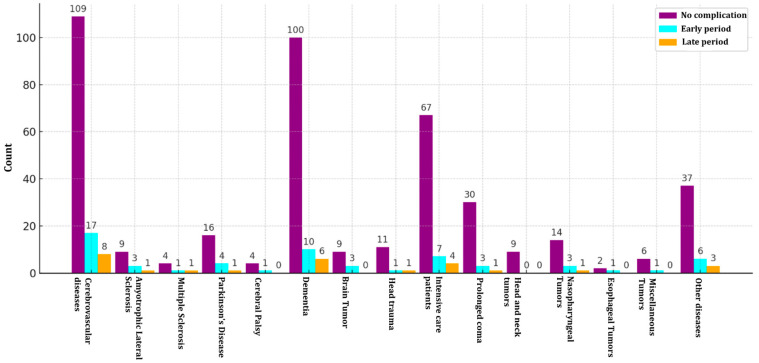
Distribution of early and late minor complications according to disease groups in patients undergoing percutaneous endoscopic gastrostomy (PEG).

**Figure 4:**
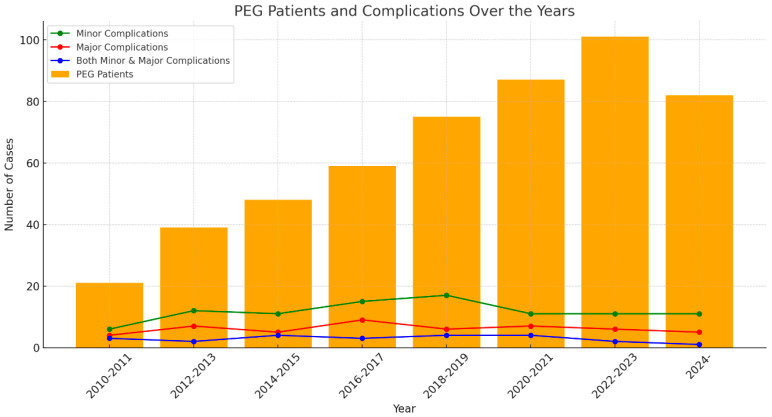
Peg patients and complications over the years.

When examining the distribution of the timing of minor complications (early and late) in patients undergoing PEG according to disease groups: 39 patients had complications in the early period (12.7%) (<2 weeks), while 17 patients had complications in the late period (5.5%) (between 2 weeks and 3 months). In patients with MS, early and late complication rates (16.6%) were found to be equal. For brain tumour patients, the early complication rate was determined to be 25%, one of the highest values in this group. In CP patients, the early complication rate was 20%, while no complications were observed in the late period. In the group of patients with decreased consciousness, the early complication rate in long-term coma patients was found to be 8.9%, and the late complication rate was 2.9%. In intensive care, early and late complication rates were calculated as 9.0% and 5.1%, respectively. In head trauma patients, early and late complication rates were equal to 7.7%. In the malignancy group, no early or late complications developed in patients with head and neck tumours. The early complication rate in nasopharyngeal tumour patients was determined to be 16.7%, with a late complication rate of 5.5%. In patients with oesophageal cancer, the early complication rate was 33%, one of the highest values in the study. No late complications were observed (Figure 3). In patients in the other unclassified diseases group, the early complication rate was found to be 13.1%, while the late complication rate was 6.5%. In our study, there was no statistically significant difference between early and late complications based on disease (*p* > 0.05) (Table 3). The distribution of PEG patients and complications over the years is summarized in Figure 4.

Regarding the reasons for changing the PEG tube, replacement according to the cycle accounted for the highest percentage and other reasons, including self-removal and functional abnormalities, were noted. Temporary intolerance was detected in six patients following tube placement. Following conservative treatment, PEG tolerance was achieved after 2–3 weeks, and it was started to be used for nutrition. There was no need for PEG removal. The average duration of tube replacement was 6 to 12 months (68%), more than 12 months (21%), and less than 6 months (11%). All PEG tube exchanges were performed under endoscopic observation. Particularly, as a method for removing the tube when exchanging the bumper-type PEG, pulling and removing through the gastrostomy hole (60%) was the most commonly used, followed by endoscopic removal after excision of the PEG tube (40%).

## 4. Discussion

PEG tube placement is an effective and safe means of providing long-term nutritional support to patients who cannot tolerate oral intake, with an estimated annual incidence of 160,000 to 200,000 cases in the United States [6]. As a minimally invasive procedure, PEG tube placement has a wide spectrum of indications. Neurological diseases affecting swallowing function, head and neck cancer patients undergoing radiation or chemotherapy, elderly patients with difficulty swallowing, and patients suffering from complications related to severe malnutrition or dysphagia constitute a significant portion of those requiring PEG.

In our study, 59.6% of patients with PEG placement were in the neurological disease group. Among neurological diseases, cerebrovascular disease (stroke) and dementia were the first two indications. The group of diseases with decreased levels of consciousness was the second-largest indication group, while malignancies ranked third. However, there are differences in the literature regarding the indications for PEG.

In the study conducted by Deza et al. [5], the most common indication was dementia (31.5%), followed by stroke (18.8%) and neuromuscular pathology (16.4%). Turan et al. [4] and Vujasinovic et al. [14] reported that the majority of patients with PEG indications were due to neurological causes, most of which were strokes. Lee et al. [15] found that the most common indications for traction PEG placement in 1625 patients were stroke (31.6%) and malignancy (18.9%). Peveling-Oberhag [13] reported that the largest portion of PEG indications were oncological patients [11], particularly those with head, neck, or oesophageal tumours (55%) fitted with PEG before starting radiotherapy, while the second largest patient group for PEG (19.8%) had neurological disorders (stroke, neurodegenerative disease, and dementia). Additionally, Schneider et al. [16] reported that malignancies were more common in PEG indications than in neurological patients. We believe that this difference may be attributed to the effects of hospital conditions, patient populations, and hospital locations. For example, during our study period, most of the intensive care units in our hospital were serving stroke patients, and the absence of an oncology clinic for an extended period may explain the low rate of malignant diseases in our study.

In the literature, the incidence of PEG-related complications varies between 13.2% and 50.1% [15,16,17,18]. In our study, we found that the minor complication rate was 17.1%, and the major complication rate was 9.5%. The most common minor complications were peristomal infection (5.2%) and bleeding from the catheter edge (3.8%), while the most common major complications were buried barrier syndrome (3.8%) and aspiration pneumonia (2.3%). The total complication rate for PEG placement was 13.2% (215 of 1625) in Lee et al.’s [15] study. The most common complication was fever without evident infection (3.5%). The remaining PEG complications included peristomal infection (3.4%), aspiration pneumonia (1.5%), and bleeding (1.2%).

Peveling-Oberhag et al. [13] detected complications in 95 patients (16.5%) in their study of 576 patients, with 11.8% being minor and 4.7% major. The most common was peristomal local infections, while the second most common was PEG dislocation. Stenberg et al. [19] reported in their series of 389 cases that the most common minor complications were tube dislocation (49%) and local infection (30%), while the most common major complications were aspiration pneumonia (74%) and organ damage (9%). Turan et al. [4] found the overall complication rate to be 11.2% in their study, reporting that the most common complications were peristomal infection and tube dislocation. A study by Desa et al. [5] found that 39.5% of patients experienced some type of complication, with local complications (28.5%) being more common than systemic complications (17.9%). The most common were tube rupture/dysfunction (13.9%) and bronchoaspiration (9.7%). Akici et al. [3] reported no major complications in their series of 378 patients, while they found peristomal infection (8.4%) and leakage around the catheter (5.8%) and bleeding around the catheter (2.1%) as minor complications. Kamiya et al. [18] reported that a total of 86 patients (22.2%) had complications, with 18.3% being minor and 3.9% major. PEG site infection was the most common minor complication, while bleeding was reported as the most serious complication.

In our study, contrary to the literature, we found the most common major complication to be BBS (3.8%). In other studies, the incidence of BBS was reported to be approximately 1% (0.3–2.4%). It usually occurs as a result of excessive compression between the inner tampon and the outer support, with the inner tampon moving along the stoma tract and coming out of the gastric wall. Over time, the gastric mucosa gradually covers the inner bumper, leading to catheter obstruction and significant leakage around the catheter. Therefore, it was determined that caregivers did not maintain a free distance (0.5–1 cm) between the skin and the outer tampon.

In our study, the evaluation of the timing of minor complications (early and late period) revealed complications in 39 patients in the early period (12.7%), while complications were observed in 17 patients in the late period (5.5%). However, there was no statistical difference in early- and late-period minor complications across disease groups.

Turan et al. [4] observed early complications occurring during or within 30 days of the placement procedure in 12.8% of cases. Boylan et al. [20] reported an early complication rate of 16.7%, while Pih et al. [21] reported a rate of 23.9%. A multicentre study by Sidorkiewicz et al. [22] revealed an even lower early complication rate of 5.14%, further confirming the favourable safety profile of PEG tube placement. Doğu et al. [11] reported early complications of 7.7% and late complications of 12.7% of patients. The most common complications in the early period were tube dislocation and peristomal infection, while in the late period, they were tube dislocation and tube occlusion. There was no significant difference in minor or major complications when classified as very early (less than one month), early (1–6 months), or late (>6 months) according to the time elapsed after PEG placement.

In recent studies, PEG-related mortality rates vary between 0 and 23%. Lee et al. [15] reported a mortality rate of 0.3% (5 of 1625 patients) within 48 h after the PEG procedure. The causes of death in these patients included peritonitis with septic shock, aspiration pneumonia, possible cardiac arrest, and exacerbation of pneumonia in lung cancer. Pih et al. [21] found pneumonia to be the most common cause of death among 20 patients who died within 30 days after PEG, with a mortality rate of 5%.

In our study, a total of six patients died due to the PEG procedure, resulting in a mortality rate of 1.1%. Three of these patients had transverse colon injuries, which led to fasciitis secondary to intra-abdominal faecal sepsis and multiple abscesses. Two patients developed pneumonia after the procedure, and one patient experienced cardiac arrest during the procedure. Turan et al. [4] reported a 30-day mortality rate of 9.7% after the PEG procedure in their study, but none of these were attributed to the PEG procedure.

This study has several limitations. First, due to its retrospective nature, there is a potential for data deficiencies and recording errors. Since patients who underwent PEG procedures at other institutions but later presented to our hospital were excluded, the reported complication rates may not fully reflect the general population. Additionally, some patients could not be reached or had insufficient information available, leading to the exclusion of a portion of the total patient cohort. Moreover, PEG procedures were not all performed by the same medical team, and variations among practitioners may have influenced complication rates. During follow-up, especially for discharged patients, data were obtained via phone interviews with caregivers, which may have introduced subjective reporting and recall bias.

## 5. Conclusions

In our study, neurological diseases, particularly cerebrovascular disease and dementia, were the most common indications for PEG placement, consistent with the literature. While PEG is generally a well-tolerated, minimally invasive procedure, complications remain significant. Our findings indicate that minor complications, such as peristomal infections and bleeding from the catheter site, were more frequent than major complications, with buried bumper syndrome being the most common major complication. Although the overall complication rate was consistent with the previous literature, the incidence of buried bumper syndrome was relatively higher in our study, underscoring the importance of proper post-procedure care and education for caregivers regarding tube maintenance. Early and late minor complications showed no significant difference across disease groups, reinforcing the need for regular follow-up and standardised post-procedure monitoring. The mortality rate associated with PEG placement in our study was within the reported range in the literature, with aspiration pneumonia being one of the leading causes of fatality.

Despite its risks, PEG remains a valuable intervention for maintaining nutritional support in critically ill and neurologically impaired patients. Further studies with larger cohorts and long-term follow-ups are needed to improve complication management and enhance patient outcomes.

## Figures and Tables

**Table 1:** Demographic data of 515 patients with PEG.

Descriptive Characteristics		Count	%
Gender	Male	218	42.4
	Female	297	57.6
Age	Mean ± SD. (Min.–Max.)	68.7 ± 15.2 (12–103)	

SD: standard deviation, Min.: minimum, and Max.: maximum.

**Table 2:** Analysis of the relationship between minor and major complications and diseases after PEG.

General Disease Group	Diseases	Complications								Minor Complications					Major Complications				
			None		Minor Complications		Major Complications			Peristomal Infection	PEG Site Leakage	PEG Edge Bleeding	Tube Dislodgement	Pneumoperitoneum	Bleeding	Aspiration Pneumonia	Internal Organ Injury	Buried Bumper Syndrome	Necrotizing Fasciitis
		*n*	Count	%	Count	%	Count	%	*p*	*n*	*n*	*n*	*n*	*n*	*n*	*n*	*n*	*n*	*n*
Neurological diseases	Cerebrovascular disease	134	95	70.90	25	18.70	14	10.40	0.000 **	7	4	6	4	4	2	4	1	6	1
	Amyotrophic lateral sclerosis	13	8	61.50	4	30.80	1	7.70	0.058	2	1	1	0	0	0	1	0	0	0
	Multiple sclerosis	6	4	66.70	2	33.30	0	0.00	0.414	0	1	1	0	0	0	0	0	0	0
	Parkinson’s disease	21	15	71.40	5	23.80	1	4.80	0.001 **	2	0	1	1	1	0	1	0	0	0
	Cerebral palsy	5	3	60.00	1	20.00	1	20.00	0.449	0	1	0	0	0	0	0	0	1	0
	Dementia	116	92	79.30	16	13.80	8	6.90	0.000 **	6	2	3	2	3	1	1	2	4	0
	Brain tumour	12	9	75.00	3	25.00	0	0.00	0.083	2	0	1	0	0	0	0	0	0	0
Reduced level of consciousness	Head trauma	13	11	84.60	2	15.40	0	0.00	0.013 *	0	1	0	1	0	0	0	0	0	0
	Intensive care patients	78	59	75.60	11	14.10	8	10.30	0.000 **	3	3	2	1	2	3	1	0	3	1
	Prolonged coma	34	28	82.40	4	11.80	2	5.90	0.000 **	2	1	1	0	0	1	0	0	1	0
Malignancy	Head and neck tumours	9	7	77.80	0	0.00	2	22.20	0.096	0	0	0	0	0	0	0	1	0	1
	Nasopharyngeal tumours	18	13	72.20	4	22.20	1	5.60	0.002 **	2	0	1	1	0	0	0	0	1	0
	Oesophageal cancer	3	2	66.70	1	33.30	0	0.00	0.564	0	1	0	0	0	0	0	0	0	0
	Miscellaneous	7	4	57.10	1	14.30	2	28.60	0.368	0	0	0	1	0	0	1	1	0	0
Others	Unclassified	46	28	60.90	9	19.60	9	19.60	0.000 **	1	2	3	2	1	2	3	0	4	0
Total		515	378		88		49	1.42		27	17	20	13	11	9	12	5	20	3

* *p* < 0.05 and ** *p* < 0.01. Chi-square test used.

**Table 3:** Analysis of the relationship between early and late minor complications and diseases after percutaneous endoscopic gastrostomy (PEG).

General Disease Group	Diseases	*n*	Complications						
			No		Early Period		Late Period		
			Count	%	Count	%	Count	%	*p*
Neurological diseases	Cerebrovascular Disease	134	109	81.3	17	12.7	8	6	1.000
	Amyotrophic Lateral Sclerosis	13	9	69.2	3	23.1	1	7.7	1.000
	Multiple Sclerosis—MS	6	4	66.8	1	16.6	1	16.6	0.521
	Parkinson’s Disease	21	16	76.2	4	19.0	1	4.7	1.000
	Cerebral Palsy	5	4	80.0	1	20.0	0	0.0	1.000
	Dementia	116	100	86.2	10	8.6	6	5.2	0.555
	Brain Tumour	12	9	75.0	3	25.0	0	0.0	0.540
Reduced level of consciousness	Head Trauma	13	11	84.6	1	7.7	1	7.7	0.521
	Intensive Care Patients	78	67	85.9	7	9.0	4	5.1	0.730
	Prolonged Coma	34	30	88.2	3	8.9	1	2.9	1.000
Malignancy	Head and Neck Tumours	9	9	100.0	0	0.0	0	0.0	1.000
	Nasopharyngeal Tumours	18	14	77.8	3	16.7	1	5.5	1.000
	Oesophageal Cancer	3	2	66.7	1	33.0	0	0.0	1.000
	Miscellaneous	7	6	85.7	1	14.3	0	0.0	1.000
Others	Unclassified	46	37	80.4	6	13.1	3	6.5	1.000

Fisher’s exact tests were used.

## Data Availability

The original contributions presented in this study are included in the article. Further inquiries can be directed to the corresponding author.

## Abbreviations

The following abbreviations are used in this manuscript:

PEG	Percutaneous endoscopic gastrostomy
PD	Parkinson’s disease
CP	Cerebral palsy
ALS	Amyothrophic lateral sclerosis
MS	Multiple sclerosis
